# Ecological Effects of Daily Antiseptic Treatment on Microbial Composition of Saliva-Grown Microcosm Biofilms and Selection of Resistant Phenotypes

**DOI:** 10.3389/fmicb.2022.934525

**Published:** 2022-06-30

**Authors:** Xiaojun Mao, Andreas Hiergeist, David L. Auer, Konstantin J. Scholz, Denise Muehler, Karl-Anton Hiller, Tim Maisch, Wolfgang Buchalla, Elmar Hellwig, André Gessner, Ali Al-Ahmad, Fabian Cieplik

**Affiliations:** ^1^Department of Conservative Dentistry and Periodontology, University Hospital Regensburg, Regensburg, Germany; ^2^Institute of Clinical Microbiology and Hygiene, University Hospital Regensburg, Regensburg, Germany; ^3^Department of Dermatology, University Hospital Regensburg, Regensburg, Germany; ^4^Department of Operative Dentistry and Periodontology, Center for Dental Medicine, University of Freiburg, Freiburg im Breisgau, Germany

**Keywords:** chlorhexidine, cetylpyridinium chloride, antiseptic, biofilm, resistance, biocide, antibiotic, dysbiosis

## Abstract

Antiseptics are widely used in dental practice and included in numerous over-the-counter oral care products. However, the effects of routine antiseptic use on microbial composition of oral biofilms and on the emergence of resistant phenotypes remain unclear. Microcosm biofilms were inoculated from saliva samples of four donors and cultured in the *Amsterdam Active Attachment* biofilm model for 3 days. Then, they were treated two times daily with chlorhexidine digluconate (CHX) or cetylpyridinium chloride (CPC) for a period of 7 days. Ecological changes upon these multiple antiseptic treatments were evaluated by semiconductor-based sequencing of bacterial 16S rRNA genes and identification of amplicon sequence variants (ASVs). Furthermore, culture-based approaches were used for colony-forming units (CFU) assay, identification of antiseptic-resistant phenotypes using an agar dilution method, and evaluation of their antibiotic susceptibilities. Both CHX and CPC showed only slight effects on CFU and could not inhibit biofilm growth despite the two times daily treatment for 7 days. Both antiseptics showed significant ecological effects on the microbial compositions of the surviving microbiota, whereby CHX led to enrichment of rather caries-associated saccharolytic taxa and CPC led to enrichment of rather gingivitis-associated proteolytic taxa. Antiseptic-resistant phenotypes were isolated on antiseptic-containing agar plates, which also exhibited phenotypic resistance to various antibiotics. Our results highlight the need for further research into potential detrimental effects of antiseptics on the microbial composition of oral biofilms and on the spread of antimicrobial resistance in the context of their frequent use in oral healthcare.

## Introduction

At present, a wide variety of antiseptics are available as over-the-counter consumer products for daily use in oral care (van der Weijden et al., 2015; Figuero et al., 2019). The use of antiseptic mouthwashes as adjunct to mechanical removal of biofilm and use of fluorides has been recommended for certain high-risk patient populations, such as patients with intellectual disabilities (Waldron et al., 2019), patients following surgical procedures, such as periodontal or implant surgery (Solderer et al., 2019), with fixed orthodontic appliances (Pithon et al., 2015), or elderly persons who are restricted in performing tooth-brushing or other oral hygiene procedures themselves (Grönbeck Lindén et al., 2017). More recently, since the COVID-19 pandemic, antiseptic mouthwashes are also applied as preprocedural mouthrinses for potentially reducing the viral load and infectivity of SARS-CoV-2 in the oral cavity and dental aerosols (Gottsauner et al., 2020; Carrouel et al., 2021; Meister et al., 2022).

Chlorhexidine digluconate (CHX), a symmetric bis-biguanide molecule carrying two positive charges, and cetylpyridinium chloride (CPC), a monocationic quaternary ammonium compound (QAC), can be regarded as the most common antiseptics for dental professional use and as ingredients in oral care products (Jones, 1997; Haps et al., 2008; Sanz et al., 2013; van der Weijden et al., 2015; Cieplik et al., 2019a; Mao et al., 2020). While both CHX and CPC are highly effective against planktonic bacteria (Cieplik et al., 2019a; So Yeon and Si Young, 2019; Mao et al., 2020), it is well known that eradicating bacterial cells in biofilms is much more difficult than killing planktonic bacteria and usually requires the antiseptic concentrations of about 100–1,000 times higher than those required to eliminate planktonic bacteria (Ceri et al., 1999; Shani et al., 2000). Accordingly, in a classic study, Zaura-Arite et al. (2001) showed that treatment with 0.2% CHX for a clinically relevant treatment period of 1 min had some effects on the outer layers of biofilms formed *in situ* for 48 h, but did not affect their inner layers. Likewise, a previous study by our group showed that a single treatment with CHX (0.1 or 0.2%) or CPC (0.05 or 0.1%) on 72 h saliva-grown microcosm biofilms resulted in colony-forming unit (CFU) reductions of only less than 1 log_10_ step (Schwarz et al., 2021). The biofilm matrix may be the main cause for this low antibacterial efficacy acting as a diffusion barrier for positively charged antiseptics such as CHX or CPC (Jakubovics et al., 2021). Therefore, it seems reasonable that bacteria in deeper layers of biofilms will be exposed to subinhibitory antiseptic concentrations upon application of antiseptic-containing mouthwash (Cieplik et al., 2019a; Mao et al., 2020; Schwarz et al., 2021; Muehler et al., 2022). Previous studies have shown that repeated exposure to subinhibitory concentrations of CHX or CPC *in vitro* may lead to phenotypic adaptation of bacteria to these antiseptics (Kitagawa et al., 2016; Cieplik et al., 2019a; Verspecht et al., 2019; Mao et al., 2020; Schwarz et al., 2021; Auer et al., 2022). Furthermore, selection pressure due to antiseptic treatment may lead to selection of antibiotic-resistant strains (Wand et al., 2017; Verspecht et al., 2019). Accordingly, we recently analyzed the transcriptomic stress response following sublethal treatment of *Streptococcus mutans* with CHX by RNA-sequencing and found considerable numbers of genes and pathways significantly upregulated or downregulated (Muehler et al., 2022). Particularly, upregulation of pathways related to stress response, increased biofilm formation, and regulation of membrane-transporters such as ATP-binding cassettes (ABC) may be linked to development of (cross-)resistances (Muehler et al., 2022).

Despite those concerns about limited antibacterial efficacy and potential risks of resistance, it is also not entirely understood which ecological changes in microbial composition of oral biofilms are induced by the routine use of antiseptic mouthwashes (Al-Kamel et al., 2019; Bescos et al., 2020a; Chatzigiannidou et al., 2020; Brookes et al., 2021; Zayed et al., 2022). Two recent studies have shown that antiseptic treatment of *in vitro* biofilms affected their microbial composition and may potentially result in ecological shifts toward increased abundance of pathobionts (Chatzigiannidou et al., 2020; Zayed et al., 2022).

Therefore, the aim for this study was first, to investigate ecological changes in mature saliva-grown microcosm biofilms upon two times daily application of CHX and CPC for a period of 7 days, and second, to evaluate, whether suchlike multiple application of CHX or CPC selects for resistant phenotypes.

## Materials and Methods

### Test Substances

Chlorhexidine digluconate (CHX; Sigma C9394) and cetylpyridinium chloride (CPC; Merck 6,002,006; both: Merck, Darmstadt, Germany) were used as antiseptics in the present study. CHX and CPC were both dissolved in dH_2_O and diluted to the respective treatment concentrations (0.1% and 0.2% for CHX, 0.05% and 0.1% for CPC).

### Saliva Collection

Four healthy volunteers (age range: 30–32 years) with no untreated dental caries, periodontitis, or other oral diseases, and no intake of antibiotics within the past 3 months volunteered for collection of saliva. Written informed consents were obtained after a detailed description of the study outline. The study protocol had been approved by the internal review board of the University of Regensburg (ref. 17-782_1-101).

The sampling was performed as described earlier (Schwarz et al., 2021). Unstimulated saliva was collected using the spitting method (Navazesh and Christensen, 1982) with the volunteers not having consumed anything except water on the respective day. The volunteers were asked to let saliva gather on the bottom of their mouth and spit into a tube every 30 s for a total period of 10 min. For separating aggregated bacteria, the collected saliva was vortexed (REAX top, Heidolph Instruments, Schwabach, Germany) for 10 s, placed in an ultrasonic water-bath chamber (Sonorex Super RK 102 H, Bandelin, Berlin, Germany; 35 kHz) for 2 min, and vortexed again for 10 s. Afterwards, saliva was divided into two aliquots, i.e., 2 ml was used for immediate biofilm inoculation and 50 μl was used as a baseline sample for 16S rRNA sequencing. For baseline samples, microbial nucleic acids were immediately stabilized by mixing 50 μl of the saliva 1:2 with magic PBI microbiome preservation buffer (microBIOMix GmbH, Regensburg, Germany). Stabilized samples were stored at –80°C until further processing.

### Inoculation and Culture of Saliva-Derived Microcosm Biofilms

Biofilms were cultured using the so-called *Amsterdam Active Attachment* (AAA) biofilm model, which is based on active attachment of the bacteria to a substrate. The AAA model consists of a custom-made stainless-steel lid with 24 clamps, which contain the respective substrates, and fits on top of a 24-well polystyrene microtiter plate, thus allowing 24 individual biofilms to form (Exterkate et al., 2010; Cieplik et al., 2019b; Schwarz et al., 2021). For the present study, hydroxyapatite (HAP) disks (9.5 mm diameter, 2 mm thickness; Clarkson Chromatography Products, South Williamsport, PA, United States) were used as a substrate in the AAA model. As a basal nutrient broth, the complete saliva broth as described by Pratten et al. (1998) was modified by adding sucrose (final concentration: 0.1%) for mimicking caries-associated conditions (caries broth; CB), as described earlier (Schwarz et al., 2021). For inoculation of the biofilms, 800 μl of the collected saliva was mixed with 40 ml CB and vigorously vortexed. Subsequently, 1.5 ml was added per each well of a 24-well plate (Corning^®^ Costar^®^, Corning, NY, United States). The steel lids containing HAP disks were placed upon, and the AAA models were incubated anaerobically (80% N_2_, 10% CO_2_, 10% H_2_) at 37°C in a microincubator (MI23NK, SCHOLZEN Microbiology Systems, St. Margrethen, Switzerland) for 8 h thus allowing initial attachment to the HAP disks. After this initial attachment period, the lids containing the HAP disks were carefully moved up and down to remove loosely bound bacteria and transferred to 24-well plates containing fresh CB. Medium was refreshed again in the same way after 24 and 48 h of culture.

### Two Times Daily Treatment of Saliva-Grown Microcosm Biofilms

After 72 h of culture, the biofilms were treated by placing the steel lid containing the HAP disks in a new 24-well plate containing either 0.1% CHX, 0.05% CPC, or 0.9% NaCl as a negative control for a treatment period of 5 min. Subsequently, the steel lid was placed in a new 24-well plate containing 0.9% NaCl to carefully wash the biofilms. This washing procedure was performed two times. Then, the steel lid was finally placed back onto a new 24-well plate containing fresh CB and incubated again anaerobically at 37°C. The biofilms were treated daily at 8 am and 4 pm for a period of 7 days resulting in 14 treatments. For each of the four donors, eight biofilms formed on separate HAP disks were used for treatment with 0.1% CHX, 0.05% CPC, or 0.9% NaCl each.

### Harvesting of the Biofilms

After 7 days of treatment with CHX, CPC, or NaCl two times daily, all biofilms were harvested by carefully removing the HAP disks from the lids using sterile forceps and transferring them to 5 ml Eppendorf tubes containing 1 ml of phosphate-buffered saline (PBS; Biochrom, Berlin, Germany). Biofilm dispersal was ensured by vortexing for 10 s, placing in an ultrasonic water-bath chamber (35 kHz) for 10 min, and vortexing again for 10 s, and confirmed visually. From those harvested samples, 50 μl was immediately stabilized by mixing with 250 μl magic PBI microbiome preservation buffer and stored at –80°C until further processing. The remaining 750 μl was immediately used for culture-based analysis.

### Extraction of Nucleic Acids and Semiconductor-Based Sequencing of Bacterial 16S rRNA Genes

Extraction of nucleic acids and semiconductor-based sequencing of bacterial 16S rRNA genes was performed, as described previously (Schwarz et al., 2021). First, pre-lysis of microbial cells was performed by mechanical cell disruption using repeated bead beating. Therefore, the total volumes of 150 μl (inoculum samples) or 300 μl (biofilm samples) stabilized sample material, respectively, were added into lysing matrix Y tubes (MP Biomedicals, Eschwege, Germany) and further processed in the TissueLyser II instrument (Qiagen, Hilden, Germany) at 60 Hz for 3 × 1 min. Nucleic acids were purified from total crude cell extracts using the MagNA Pure 96 instrument (Roche Diagnostics, Mannheim, Germany). Quantification of total nucleic acids was carried out by means of the NanoDrop™ 1000 spectrophotometer (Thermo Fisher Scientific, Darmstadt, Germany).

Copy numbers of bacterial 16S rRNA genes were quantified in nucleic acid extracts by qRT-PCR, as described earlier (Hiergeist et al., 2016). Subsequently, V1–V3 hypervariable regions of bacterial 16S rRNA genes were amplified from a total of 1e + 7 bacterial 16S rDNA copies for each sample using primer S-D-Bact-0008-c-S-20 containing a 10-bp barcode sequence and IonTorrent-specific sequencing adaptor A, and S-D-Bact-0517-a-A-18 containing a 3’-P1 adapter sequence using the Platinum II Taq Hot-Start DNA Polymerase (Thermo Fisher Scientific). After 30 PCR cycles, amplicons were purified two times with a 0.8 bead to DNA ratio using MagSi-NGS*^PREP^* Plus beads (Steinbrenner Laborsysteme, Wiesenbach, Germany). Copy numbers of amplicons containing sequencing-adaptors were determined using the KAPA Library Quantification IonTorrent Kit (Roche Diagnostics) and pooled to equimolar amplicon concentrations of each sample. A total of 120 attomol of the final library pool was subjected to isothermal amplification with the IonChef instrument before running 1350 flow cycles during high-throughput sequencing on an Ion Torrent™ S5 Plus machine (Thermo Fisher Scientific).

### Sequence Processing and Identification of Amplicon Sequence Variants

First, amplification primer and adapter sequences and low-quality bases were removed using cutadapt 3.5 and Trimmomatic 0.39. Cutadapt was also used for demultiplexing of filtered reads allowing no errors. All subsequent analyses were conducted with R 4.1.2. Here, the resulting reads (19,573 ± 7,048) were subjected to denoising sequencing data and generation of Amplicon Sequence Variants (ASVs) using dada2 (version 1.16). An unrooted phylogenetic tree was calculated with FastTree 2.1 after sequence alignment with DECIPHER 2.20 for later calculation of UniFrac distances with the phyloseq package. The IDTAXA algorithm and the All-Species Living Tree Project (LTP) reference database 12.2021 release was used for taxonomic classification of ASVs. Significantly altered taxa between groups were assessed with the linear discriminant analysis (LDA) effect size (LEfSe) method which is included within the microbiomeMarker R package (Cao, 2021). All plots were generated using the ggpubr 0.4 package.

### Colony-Forming Units Assay, Identification of Antiseptic-Resistant Phenotypes, and Evaluation of Antibiotic Susceptibility

Immediately after harvesting the biofilms, 10-fold serial dilutions (10^–1^ to 10^–7^) were prepared in PBS and aliquots (180 μl) were plated on Schaedler blood agar plates for determination of total CFU following the two times daily treatment with CHX, CPC, or NaCl over 7 days, and incubated anaerobically at 37°C for 72 h. Afterward, CFU were evaluated.

Furthermore, aliquots (180 μl) from the lowest dilution steps (10^–1^ to 10^–4^) were plated on Schaedler blood agar plates containing 0.1 or 0.2% CHX for CHX-treated and NaCl-treated biofilms or containing 0.05 or 0.1% CPC for CPC-treated and NaCl-treated biofilms in order to investigate for antiseptic-resistant phenotypes. After anaerobic incubation at 37°C for 72 h, the plates were evaluated for growth of antiseptic-resistant phenotypes. Those colonies were first discriminated according to their respective colony morphology and separated by sub-culturing on fresh agar plates. These colonies were identified at the species level by means of matrix-assisted laser desorption/ionization time-of-flight mass spectrometry (MALDI-TOF MS) employing a Microflex mass spectrometer and BioTyper analysis software (both from Bruker, Billerica, MA, United States), as described earlier (Cieplik et al., 2020).

The antiseptic-resistant isolates (despite Enterobacteriaceae) were analyzed for their antibiotic susceptibilities by means of the ETEST^®^ method (bioMérieux, Marcy l’Etoile, France). In brief, suspensions (McFarland 1.0) of *Capnocytophaga*, *Fusobacterium*, and *Veillonella* spp. were inoculated on Brucella blood agar (bioMérieux), *Campylobacter* spp. on Mueller-Hinton (MH) agar with horse blood (Oxoid, Wesel, Germany), and *Neisseria* spp. on MH blood agar (Oxoid), and the ETEST^®^ strips were placed on the agar plates. Results were evaluated following incubation for 48 h at 37°C under microaerophilic (*Campylobacter* spp.) or anaerobic (*Capnocytophaga*, *Fusobacterium*, *Veillonella*, and *Neisseria* spp.) conditions. Using ETEST^®^, the following antibiotics were investigated: penicillin G, ampicillin, amoxicillin/clavulanic acid, piperacillin/tazobactam, imipenem, ceftriaxone/cefotaxime, ceftazidime, ciprofloxacin, erythromycin, clindamycin, tetracycline, and metronidazole.

Antibiotic susceptibilities of Enterobacteriaceae were tested using the BD Phoenix NMIC panel (Becton Dickinson, Sparks, MD, United States) according to the instructions of the manufacturer for the following antibiotics: ampicillin/amoxicillin, amoxicillin/clavulanic acid, piperacillin, piperacillin/tazobactam, imipenem, meropenem, ertapenem, aztreonam, cefuroxime, cefoxitin, ceftriaxone/cefotaxime, ceftazidime, cefepime, ciprofloxacin, levofloxacin, gentamicin, amikacin, tobramycin, fosfomycin, and trimethoprim/sulfamethoxazole. Interpretation of the results of both methods was done according to the EUCAST (European Committee on Antimicrobial Susceptibility Testing) 12.0 guidelines, and susceptibility was determined as susceptible (S), intermediate (I), or resistant (R), whereby non-species related breakpoints were used if no species-specific breakpoints were available.

## Results

### Microbial Composition of Saliva-Grown Microcosm Biofilms and Ecological Effects of Daily Treatment With Chlorhexidine Digluconate or Cetylpyridinium Chloride

**FIGURE 1:**
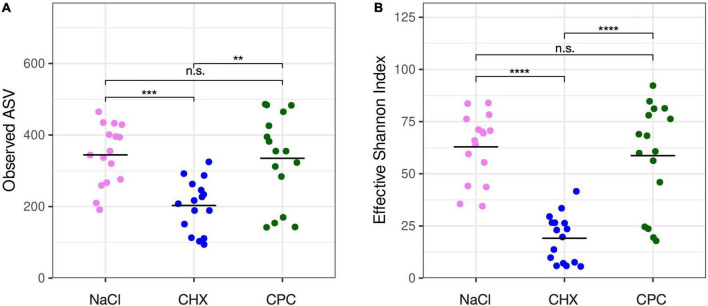
Alpha-diversity of the biofilms as shown by observed ASVs **(A)** and effective Shannon index **(B)**. CHX-treated biofilms showed significantly lower alpha-diversity as compared to NaCl-treated or CPC-treated biofilms with no significant differences between the latter. Significance levels are indicated by asterisks: **p ≤ 0.01, ***p ≤ 0.001, ****p ≤ 0.0001.

A total of 4,036 (mean 294 ± 115 per sample) amplicon sequence variants (ASVs) were detected by high-throughput sequencing of V1–V3 variable regions of bacterial 16S rRNA genes. Alpha-diversity represented by the number of detected ASVs (Figure 1A) and the Effective Shannon Index (Figure 1B) was significantly lower in CHX-treated biofilms (mean 203 ± 72 ASVs; mean 19 ± 11 Shannon) as compared to NaCl-treated (mean 344 ± 84 ASVs; mean 63 ± 16 Shannon) or CPC-treated biofilms (mean 335 ± 125 ASVs; mean 59 ± 25 Shannon) with no significant differences between the latter.

**FIGURE 2:**
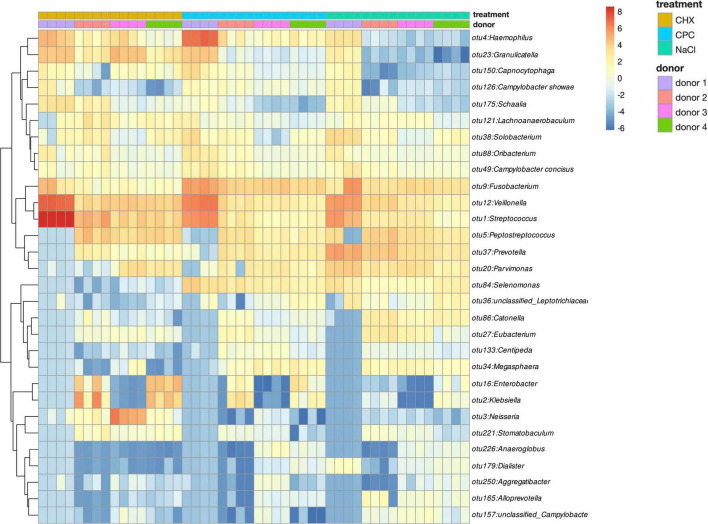
Heatmap of ASV abundance on genus level for NaCl-, CHX-, and CPC-treated biofilms from all four donors.

**FIGURE 3:**
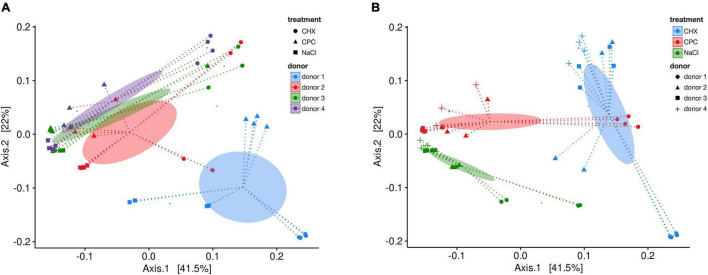
Beta-diversity analysis by principal coordinate analysis (PCoA) of weighted UniFrac distances for NaCl-, CHX-, and CPC-treated biofilms from all four donors. Ellipses indicate the 95% confidence interval of group centroids summarized for donors **(A)** or treatments **(B)**. Depicted are coordinates 1 and 2, which explained 63.5% of the total variance. (Adonis NaCl vs. CHX: *R*^2^ = 0.42, p.adj = 0.003, NaCl vs. CPC: *R*^2^ = 0.23, p.adj = 0.003, CHX vs. CPC: *R*^2^ = 0.36, p.adj = 0.003).

Figure 2 depicts a heatmap of ASV abundance on genus level for NaCl-treated, CHX-treated, and CPC-treated biofilms from all four donors. The biofilms show a diverse microbial composition with *Streptococcus*, *Veillonella*, *Fusobacterium*, *Haemophilus*, and *Granulicatella* spp. being the most abundant. The heatmap clearly depicts the differences in microbial composition between the biofilms from different donors as well depending on the respective treatment. Accordingly, beta-diversity based on weighted UniFrac distances showed clear clustering depending on the donor (Figure 3A; Adonis *R*^2^ = 0.25, p.adj = 0.001) and also regarding their respective treatment (Figure 3B; Adonis *R*^2^ = 0.41, p.adj = 0.001).

**FIGURE 4:**
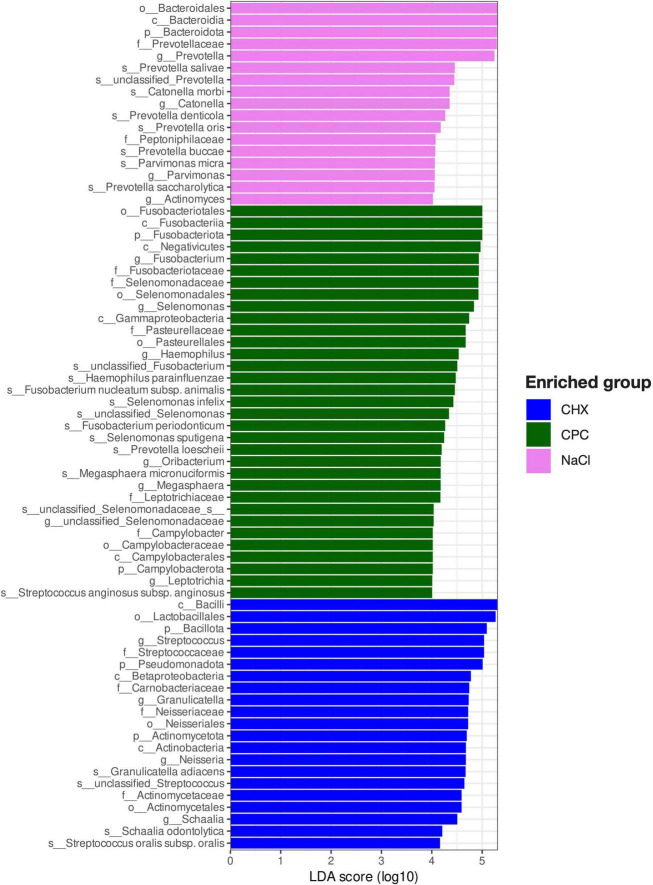
Discriminatory ASVs for biofilms treated with NaCl, CPC, or CHX, respectively, as identified by linear discriminant analysis (LDA) effect size (LEfSe). ASVs exhibiting LDA-score ≥ 4 and adjusted *p*-values < 0.01 are shown.

Several ASVs were found to be discriminatory between the biofilms treated by CHX, CPC, or NaCl, as revealed by LefSE (Figure 4). Accordingly, the CHX-treated biofilms were characterized by the enrichment of several ASVs within the orders Lactobacillales (mainly *Streptococcus* and *Granulicatella* spp.), Neisseriales (mainly *Neisseria* spp.), and Actinomycetales (mainly *Schaalia* spp.). In contrast, CPC-treated biofilms were enriched by several ASVs within the orders Fusobacteriales (mainly *Fusobacterium* and *Leptotrichia* spp.), Selenomonadales (mainly *Selenomonas* spp.), Pasteurellales (mainly *Haemophilus* spp.), and Campylobacterales, as well as *Oribacterium* and *Prevotella loescheii*. Furthermore, both CHX- and CPC-treated biofilms were characterized by a loss of several *Prevotella*, *Catonella*, and *Parvimonas* spp. as compared to the NaCl-treated biofilms.

### Colony-Forming Units Assay and Identification of Antiseptic-Resistant Phenotypes

**FIGURE 5:**
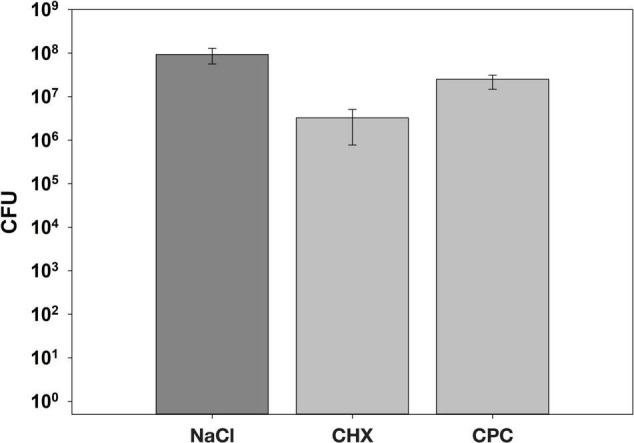
The CFU results following two times daily treatment with either 0.9% NaCl, 0.1% CHX, or 0.05% CPC for a period of 7 days. All results are depicted as medians, 1st and 3rd quartiles from eight individual biological replicates on a log_10_-scaled ordinate.

Figure 5 shows the CFU assay results. Biofilms treated with NaCl exhibited median CFU numbers of 9.3 × 10^7^ CFU, while CHX- or CPC-treated biofilms showed 3.3 × 10^6^ or 2.5 × 10^7^ CFU, respectively, resulting in CFU-reductions of 1.5 log_10_ steps for CHX and 0.6 log_10_ steps for CPC as compared to the biofilms treated with NaCl.

Table 1 summarizes the antiseptic-resistant phenotypes as identified by MALDI-TOF MS along with their respective antibiotic susceptibilities as tested using ETEST^®^ or by means of the BD Phoenix NMIC panel. In each donor, at least one taxon could be isolated that was able to grow on the antiseptic-containing agar plates. In donor 1, *Capnocytophaga sputigena* was found on the CHX-containing agar plate from the NaCl-treated biofilm and was susceptible to all tested antibiotics, while *Campylobacter showae*, which was isolated on the CPC-containing agar plate from both NaCl- and CPC-treated biofilms presented resistance to ciprofloxacin and piperacillin/tazobactam. Moreover, *Campylobacter showae*, which was isolated on the CPC-containing agar plate from CPC-treated biofilms, showed additional resistance to Penicillin G. In donor 2, only taxa identified as *Klebsiella oxytoca or Raoultella* sp. were isolated on both CHX- and CPC-containing agar from the corresponding antiseptic-treated biofilms. Both *Klebsiella oxytoca* and *Raoultella* sp. isolates showed resistance to ampicillin/amoxicillin, piperacillin, and fosfomycin. The highest number of antiseptic-resistant phenotypes was isolated from donor 3. *Capnocytophaga sputigena* (susceptible to all tested antibiotics) and *Capnocytophaga gingivalis* (susceptible to all tested antibiotics) were isolated on the CHX-containing agar plate from the NaCl-treated biofilms, while *Fusobacterium* sp. (resistant to penicillin G, ampicillin and ciprofloxacin), *Veillonella rogosae* (resistant to clindamycin and intermediate resistant to ceftazidime), and *Campylobacter curvus* (intermediate resistant to ciprofloxacin) were isolated on the CPC-containing agar plate from the NaCl-treated biofilm. Additionally, *Neisseria perflava* (resistant to penicillin G, ampicillin and ceftriaxone/cefotaxime) was isolated from the CHX-treated biofilm and *Veillonella rogosae* (resistant to clindamycin) was isolated from the CPC-treated biofilm. In donor 4, two strains identified as *Klebsiella oxytoca* or *Raoultella* sp. were collected from NaCl- or CPC-treated biofilms and isolated from CHX- or CPC-containing agar. Both showed resistance to ampicillin/amoxicillin, piperacillin, and fosfomycin. One taxon identified to be from the *Enterobacter cloacae*-complex was isolated on the CHX-containing agar originating from the CHX-treated biofilms growing and exhibited resistance to ampicillin/amoxicillin, amoxicillin/clavulanic acid, piperacillin, piperacillin/tazobactam, and cefazolin.

**TABLE 1:** Antiseptic-resistant phenotypes isolated from the biofilms and evaluation of antibiotic susceptibilities.

A. Antiseptic-resistant phenotypes and evaluation of their antibiotic susceptibilities by means of ETEST^®^															

				Beta-lactams							Fluoroquinolones	Macrolides and Lincosamides		Tetracycline	Nitroimidazole
			
Donor	Treatment group	Growth on plate	Identification by MALDI-TOF MS	Penicillin G	Ampicillin	Amoxicillin/clavulanic acid	Piperacillin/tazobactam	Imipenem	Ceftriaxone Cefotaxime	Ceftazidime	Ciprofloxacin	Erythromycin	Clindamycin	Tetracycline	Metronidazole
1	NaCl	0.01% CHX	*Capnocytophaga sputigena*	0.002 S	0.016 S	0.016 S	0.016 S	0.002 S	0.006 S	0.032 S	0.003 S	0.125 –	0.006 –	0.023 –	0.047 –
1	NaCl	0.01% CPC	*Campylobacter showae*	2 S	0.032 S	0.032 S	24 **R**	0.094 S	0.064 S	0.125 S	≥ 32 **R**	4 S	0.25 –	0.19 –	≥ 256 –
1	CPC	0.01% CPC	*Campylobacter showae*	4 **R**	0.094 S	0.032 S	48 **R**	0.125 S	0.094 S	0.25 S	≥32 **R**	4 S	0.75 –	0.75 –	≥256 –
3	NaCl	0.01% CHX	*Capnocytophaga sputigena*	0.002 S	0.016 S	0.016 S	0.016 S	0.002 S	0.002 S	0.016 S	0.006 S	0.125 –	0.016 –	0.023 –	0.016 –
3	NaCl	0.01% CHX	*Capnocytophaga gingivalis*	0.016 S	0.023 S	0.023 S	0.016 S	0.032 S	0.5 S	0.38 S	0.006 S	≥256 –	0.006 –	0.047 –	3 S
3	NaCl	0.01% CPC	*Fusobacterium* sp.	≥32 **R**	≥256 **R**	0.016 S	0.19 S	0.047 S	1 S	3 S	0.75 **R**	4 –	0.75 –	0.19 –	0.016 S
3	NaCl	0.01% CPC	*Veillonella rogosae*	0.125 S	0.094 S	0.094 S	0.75 S	0.125 S	0.25 S	8 I	0.032 S	16 –	≥256 **R**	0.064 –	0.38 S
3	NaCl	0.01% CPC	*Campylobacter curvus*	1 S	0.032 S	0.032 S	24 S	0.19 S	0.032 S	0.38 S	0.047 I	2 S	1.5 –	0.19 S	6 –
3	CHX	0.05% CHX	*Neisseria perflava*	6 **R**	1.5 **R**	1.5 S	1 S	0.75 S	0.19 **R**	*N*	0.012 S	32 -	32 –	1.5 –	256 –
3	CPC	0.05% CPC	*Veillonella rogosae*	0.25 S	0.19 S	0.19 S	0.19 S	0.064 S	0.25 S	8 I	0.047 S	24 -	≥ 256 **R**	0.125 –	0.38 S

B. Antiseptic-resistant phenotypes and evaluation of their antibiotic susceptibilities by means of the BD Phoenix NMIC panel.																							

				Beta-lactams													Fluoroquinolones		Aminoglycosides			Others	
Donor	Treatment group	Growth on plate	Identification by MALDITOF-MS	Ampicillin/amoxicillin	Amoxicillin/clavulanic acid	Piperacillin	Piperacillin/tazobactam	Imipenem	Meropenem	Ertapenem	Aztreonam	Cefuroxime	Cefoxitin	Ceftriaxone/Cefotaxime	Ceftazidime	Cefepime	Ciprofloxacin	Levofloxacin	Gentamicin	Amikacin	Tobramycin	Fosfomycin	Trimethoprim/sulfamethoxazole
2	CHX	0.01% CHX	*Klebsiella oxytoca*/ *Raoultella* sp.	>8 **R**	≤2/2 S	8 **R**	≤4/4 S	0.5 S	≤0.125 S	≤0.25 S	≤1 S	≤2 I	≤4 S	≤0.5 S	≤0.5 S	≤1 S	≤0.25 S	≤0.5 S	≤1 S	≤4 S	≤1 S	>128 **R**	≤1/19 S
2	CPC	0.05% CPC	*Klebsiella oxytoca*/ *Raoultella* sp.	> 8 **R**	≤ 2/2 S	8 **R**	≤ 4/4 S	0.5 S	≤ 0.125 S	≤0.25 S	≤ 1 S	≤2 I	≤ 4 S	≤0.5 S	≤ 0.5 S	≤1 S	≤ 0.25 S	≤0.5 S	≤ 1 S	≤4 S	≤ 1 S	> 128 **R**	≤ 1/19 S
4	NaCl	0.01% CHX	*Klebsiella oxytoca*/ *Raoultella* sp.	>8 **R**	≤2/2 S	≤4 **R**	≤4/4 S	0.5 S	≤0.125 S	≤0.25 S	≤1 S	≤2 I	≤4 S	≤0.5 S	≤0.5 S	≤1 S	≤0.25 S	≤0.5 S	≤1 S	≤4 S	≤1 S	≤16 S	≤1/19 S
4	CHX	0.05% CHX	*Enterobacter cloacae* complex	>8 **R**	≤32/2 S	16 **R**	≤16/4 S	≤0.25 S	≤0.125 S	≤0.25 S	≤1 S	*N*	>16 **R**	≤0.5 S	1 S	≤1 S	≤0.25 S	≤0.5 S	≤1 S	≤4 S	≤1 S	≤16 S	≤1/19 S
4	CPC	0.05% CPC	*Klebsiella oxytoca*/ *Raoultella* sp.	>8 **R**	≤2/2 S	≤4 **R**	≤4/4 S	≤0.25 S	≤0.125 S	≤0.25 S	≤1 S	≤2 I	≤4 S	≤0.5 S	≤0.5 S	≤1 S	≤0.25 S	≤0.5 S	≤1 S	≤4 S	≤1 S	≤16 S	≤1/19 S

*(A) The first line shows the respective ETEST^®^ result [μg/mL], while the second line gives the interpretation according to EUCAST 12.0 (S, susceptible; I, intermediate; **R**, resistant; –, no breakpoint given). N, not tested.*

*(B) The first line shows the respective MIC [μg/mL], while the second line gives the interpretation according to EUCAST 12.0 (S, susceptible; I, intermediate; **R**, resistant). N, not tested.*

## Discussion

Antiseptics are in widespread use in dental practice and also included in numerous over-the-counter oral care products (Haps et al., 2008; Sanz et al., 2013; van der Weijden et al., 2015), but the effects of routine antiseptic use on microbial composition of oral biofilms (Chatzigiannidou et al., 2020; Zayed et al., 2022) and on the emergence of resistant phenotypes are still unclear (Cieplik et al., 2019a; Mao et al., 2020). Therefore, this study aimed to investigate the ecological effects of daily treatment with CHX or CPC on mature saliva-grown biofilms and whether a suchlike treatment selects for resistant phenotypes.

For this purpose, microcosm biofilms were cultured from human saliva employing the so-called *Amsterdam Active Attachment* (AAA) biofilm model, as described previously (Schwarz et al., 2021). Sampling was performed from healthy donors, and a basal nutrient broth mimicking human saliva was modified by adding sucrose (Pratten et al., 1998; Cieplik et al., 2013; Schwarz et al., 2021) to provide environmental conditions leading to biofilms that resemble microbial communities in rather early stage of dysbiosis dominated by early colonizers of dental plaque (Schwarz et al., 2021). While the biofilms in this previous study showed rather low alpha-diversity and mostly growth of *Streptococcus* and *Veillonella* spp., a considerably higher alpha-diversity was found for the biofilms in the present study. This may be attributed to several factors: Once, HAP disks were used as a substrate for biofilm culture instead of glass disks as used previously (Schwarz et al., 2021). These HAP disks may better mimic dental hard tissues and might improve bacterial attachment toward the substrate (Hannig and Hannig, 2009), which is particularly crucial for the AAA model used in the present study, as it relies on the active attachment of bacteria (Exterkate et al., 2010; Cieplik et al., 2019b). Furthermore, another important aspect may be the different period for culture of the biofilms. While the biofilms were cultured only for 3 days in the previous study (Schwarz et al., 2021), here, the biofilms were cultured for 10 days in total (3 days of biofilm formation followed by 7 days of treatment with NaCl, CHX, or CPC), which may have given the more fastidious bacteria more time to establish themselves in the biofilms (Edlund et al., 2013; Kistler et al., 2015; Cieplik et al., 2019b). The microbial compositions of the biofilms were found dependent on the respective donor source, which is in line with the results from a previous study, where we found a much stronger clustering of microbial compositions of biofilms per each donor than per niche of each donor even after up to 28 days of *in vitro* culture, indicating a strong donor-driven “fingerprint” (Cieplik et al., 2019b). Similar results were reported by Chatzigiannidou et al. (2020) who also observed a strong donor-dependency regarding microbial composition of their tongue-swab-derived microcosm biofilms. Notably, here, the biofilms from one donor (donor 1) clustered particularly different from the other three donors. This may be explained by the different ethnicity of this donor (Asian, while the other three donors were Caucasian), as also shown in previous studies (Mason et al., 2013; Premaraj et al., 2020). For instance, microbial communities in saliva and subgingival biofilms were found to have distinct ethnicity profiles, and based on these results, it was even possible to identify the ethnicity of individuals from subgingival microbial signatures using a machine learning classifier (Mason et al., 2013). Furthermore, a recent metagenome-wide association study found that human genetics account for at least 10% of oral microbiome compositions between different individuals, which may also explain the stable microbial composition within one single individual over time (Liu et al., 2021).

After undisturbed culture of the biofilms for 3 days, a mouthwash was simulated two times daily using either of the tested compounds for 5 min each. The AAA biofilm model facilitates controlling treatment periods as opposed to other biofilms models, which are based on bacterial sedimentation rather than active bacterial attachment (Exterkate et al., 2010). Due to the well-known high substantivity of CHX and CPC (Elworthy et al., 1996), biofilms were washed after the 5 min treatment period to dilute potentially remaining CHX or CPC and limit potential prolonged effects of both antiseptics. The two times daily treatment with CHX or CPC reduced CFU in the biofilms only by 1.5 log_10_ or 0.6 log_10_ steps as compared to the NaCl group. These results clearly show that both antiseptics exhibited only temporary effects and could not inhibit bacterial regrowth, in line with several other studies indicating that antiseptic mouthwashes are not able to effectively limit microbial numbers, particularly when applied to mature biofilms (Cieplik et al., 2019a; Chatzigiannidou et al., 2020; Brookes et al., 2021; Schwarz et al., 2021). Therefore, it is crucial to investigate the ecological effects on the microbial composition of the surviving microbiota, which is still discussed controversially for CHX and has not been investigated so far for CPC (Al-Kamel et al., 2019; Bescos et al., 2020a; Chatzigiannidou et al., 2020; Brookes et al., 2021).

We found that treatment with CHX significantly reduced alpha-diversity in the biofilms as compared to treatment with CPC or NaCl (with no significant difference between the latter), in accordance with several other studies evaluating the effects of CHX on microbial communities *in vitro* and *in vivo* (Fernandez et al., 2017; Tribble et al., 2019; Brookes et al., 2020, 2021; Chatzigiannidou et al., 2020). Furthermore, beta-diversity showed a strong and significant ecological shift following treatment with CHX, resulting in enrichment of rather caries-associated taxa such as *Streptococcus*, *Neisseria*, *Schaalia* [genus recently created by subdivision from *Actinomyces* (Nouioui et al., 2018)], and *Granulicatella* spp. For instance, the significantly enriched species *Streptococcus oralis* subsp. *oralis* and *Schaalia odontolytica* (formerly classified as *Actinomyces odontolyticus*) have been associated with dental caries (ElSalhy et al., 2016; Da Costa Rosa et al., 2021). Bescos et al. (2020a) investigated the effects of 7-day use of a CHX mouthwash on the salivary microbiota in 36 healthy individuals. They observed an increase in the abundance of taxa from the genera *Streptococcus*, *Neisseria*, and *Granulicatella*, but a decrease of *Actinomyces* (Bescos et al., 2020a), and a significantly lower salivary pH and buffer capacity after using the CHX mouthwash for 7 days, concluding that CHX may have a significant impact on the oral microbiota, potentially favoring dental caries (Bescos et al., 2020a). Likewise, Chatzigiannidou et al. (2020) found an ecological shift toward a streptococci-dominated microbial community and increased lactate production after treating *in vitro* 14-species biofilms with CHX over 3 days for 5 min each, while they observed a contrary trend with increase in *Granulicatella* and *Fusobacterium* spp. after treating microcosm biofilms inoculated from tongue scrapings.

Interestingly, treatment with CPC had a different effect on the biofilms. Alpha-diversity was not significantly affected as compared to the NaCl-treated biofilms, but beta-diversity also revealed a significant ecological shift, resulting in enrichment of proteolytic and Gram-negative taxa such as *Fusobacterium*, *Leptotrichia*, and *Selemonas* spp. as well as *Oribacterium*, which are mainly associated with gingivitis (Diaz et al., 2016; Bryan et al., 2017; Nowicki et al., 2018).

Both antiseptic treatments led to a loss of *Prevotella* spp., which are known nitrite producers and associated with high nitrate-reduction capacity (Hyde et al., 2014; Rosier et al., 2022). Accordingly, clinical studies have shown that the use of CHX mouthwashes led to lower nitrite concentrations in saliva and plasma followed by slight increases in systolic blood pressure (Tribble et al., 2019; Bescos et al., 2020a). Although the oral microbiota is known to be highly resilient, particularly as compared to the intestinal microbiota (Wade, 2021), clinicians should be aware of potential detrimental effects of long-term use of antiseptic mouthwashes with regard to oral microbial ecology (Bescos et al., 2020b), which may potentially further perturb the commensal microbiota rather than shifting to a health-associated state (Chatzigiannidou et al., 2020). However, it needs to be considered that here the effects of “pure” antiseptics were investigated, whereas the effects of antiseptic mouthwashes seem to be strongly dependent on their respective compositions and formulations, as recently shown (Zayed et al., 2022). Also, a commercially available mouthwash comprising both CHX and CPC showed capability to even improve the microbial ecology of a 14-species biofilm *in vitro* reducing the level of pathobionts to less than 10% (Zayed et al., 2022), whereas in our study, CHX led to enrichment of rather caries-associated saccharolytic taxa and CPC led to enrichment of rather gingivitis-associated proteolytic taxa.

Despite analyzing effects on biofilm ecology, we also sought to investigate whether antiseptic treatment selects for resistant phenotypes. For this purpose, an agar dilution technique was employed, and the biofilms were plated on Schaedler agar plates containing 0.01 or 0.05% CHX or CPC, respectively. Although this method is in line with the guidelines of the Clinical Laboratory Standards Institute (CLSI) (CLSI, 2018a,b) and has also been used in earlier studies investigating antiseptics (Eick et al., 2011; Akca et al., 2016), it should be considered that the biologically available concentrations on the surface of the plates may not necessarily reflect the rather high concentrations mixed into the agar plates, due to potential interactions of the cationic antiseptics and constituents of the solid growth media (Akca et al., 2016). Thus, only qualitative, but no quantitative results (i.e., CFU numbers) are reported in this study. We found antiseptic-resistant phenotypes in biofilms from each donor, which also showed resistance to various antibiotics. As some of them were isolated from the NaCl-treated biofilms, these isolates originate from the inoculum source and could establish in the biofilms even without selection pressure due to the two times daily antiseptic treatment, supporting recent studies, which highlight the oral microbiota as a reservoir of antimicrobial resistance (AMR) genes (Jiang et al., 2018; Arredondo et al., 2020).

The isolated antiseptic-resistant phenotypes were found to be highly donor-dependent: In the biofilms from two donors (1 and 3), typical oral taxa could be isolated from the antiseptic-containing agar plates. *Capnocytophaga* spp. could be isolated from CHX-containing agar, in line with older studies reporting that *Capnocytophaga* spp. exhibited minimum inhibitory concentrations (MICs) for CHX of up to 250 μg/ml (0.025%) and were the least susceptible among all oral bacteria included in these experiments (Stanley et al., 1989; Wade and Addy, 1989). Furthermore, the earliest study probably reporting isolation of this genus was from a group of dental students following use of a 0.2% CHX mouthwash for 22 days (Davies et al., 1972; Leadbetter et al., 1979). Two *V. rogosae* strains, which were both isolated from CPC-containing agar, showed high-level resistance to clindamycin, which was also found in 55% of *Veillonella* isolates in a previous study (Teng et al., 2002). *N. perflava* isolated from CHX-treated biofilms on CHX-containing agar was found resistant to penicillin G, ampicillin, and ceftriaxone/cefotaxime, which is in line with a recent systematic review stating that antimicrobial susceptibilities in commensal as well as pathogenic *Neisseria* spp. have been increasing considerably following decades of antibiotic exposure (Vanbaelen et al., 2022). Remarkably, the MIC of 6 μg/ml found here for penicillin G is higher than all reported for commensal *Neisseria* spp. in this systematic review (Vanbaelen et al., 2022). *Campylobacter* spp. could be isolated from CPC-containing agar. The two *C. showae* isolates showed high-level resistance to ciprofloxacin and piperacillin/tazobactam, while they were sensitive to amoxicillin/clavulanic acid. Resistance to piperacillin has previously been described in poultry isolates of *Campylobacter* spp., although MICs of piperacillin/tazobactam were 16- to 32-fold lower than for piperacillin alone, they still were found quite high (around 32 μg/ml) (Griggs et al., 2009), in line with the MICs found here. In *Campylobacter* spp., resistance to fluoroquinolones like ciprofloxacin is mainly due to single point mutation(s) in the *gyrA* gene (Wieczorek and Osek, 2013; Sproston et al., 2018). An isolate of the genus *Fusobacterium*, which was obtained from CPC-containing agar, exhibited high-level resistance to penicillin G and ampicillin, which was also found previously and attributed to be due to expression of a class D beta-lactamase (Al-Haroni et al., 2008).

Apart from these typical oral taxa, Enterobacteriaceae could be isolated from the biofilms from donors 2 and 4. Although members of Enterobacteriaceae are usually considered as transient components of the oral microbiota, they have consistently been detected at low numbers in subgingival biofilm samples (Espíndola et al., 2022; Jepsen et al., 2022) and also on toothbrushes (Zinn et al., 2020). For instance, Jepsen et al. (2022) recently analyzed biofilm samples collected between 2008 and 2015 from deep periodontal pockets in 16,612 German adults diagnosed with periodontitis and found mean annual prevalence rates of 3.6% for *K. oxytoca* and 2.5% for *E. cloacae*. The fosfomycin resistance detected in two *K. oxytoca*/*Raoultella* sp. isolates may be attributable to the chromosomal *fosA* gene, which is present in the majority of genomes in *Klebsiella* spp. (Ito et al., 2017). Likewise, the resistance of all Enterobacteriaceae isolated in the present study to aminopenicillins such as ampicillin, amoxicillin, and piperacillin and, in part, to second-generation cephalosporins like cefuroxime or cefoxitin may be mainly attributed to their production of AmpC-type beta-lactamases (Meini et al., 2019). Thus, adjunctive prescription of amoxicillin, e.g., in the course of periodontal treatment, may pose the risk of overgrowth of such taxa in oral biofilms (Jepsen et al., 2022). Accordingly, Baker at al. (2019) reported recently that *K. oxytoca*, *K. pneumoniae*, and *Providencia alcalifaciens* were the only taxa found to be transcriptionally active and recoverable after long-term starvation of a saliva-derived microbial community over 100 days *in vitro*, which may explain that hospital surfaces contaminated with saliva can serve as source of outbreaks of drug-resistant Enterobacteriaceae. Although such long-term starvation does not reflect the environmental conditions in the oral cavity (Baker et al., 2019), it may be similar to long-term selective pressure due to extensive use of antiseptic mouthwashes (Cieplik et al., 2019a; Mao et al., 2020). Besides selection of intrinsically less susceptible phenotypes, adaptation to antiseptics such as CHX may also occur, which was recently linked to development of antibiotic cross-resistance to colistin in *K. pneumoniae* (Wand et al., 2017).

## Conclusion

This study shows that both CHX and CPC exhibited significant ecological effects on the microbial compositions of microcosm biofilms upon two times daily treatment for a period of 7 days. CHX led to enrichment of rather caries-associated saccharolytic taxa as compared to NaCl-treated biofilms, while CPC led to enrichment of rather gingivitis-associated proteolytic taxa. Antiseptic-resistant phenotypes could be isolated from all biofilms regardless which treatment group they belonged to. These isolates exhibited resistance to various antibiotics. Therefore, further studies are needed to elucidate implications of the widespread use of antiseptics in oral healthcare with regard to their ecological effects on oral biofilms as well as on the spread of antimicrobial resistance. Clinicians should be aware of the potential risks associated with the widespread and indiscriminate use of antiseptics and apply or prescribe them only for appropriate indications and preferably only for short periods of time.

## Data Availability Statement

The datasets presented in this study can be found in online repositories. The names of the repository/repositories and accession number(s) can be found below: NCBI – PRJEB52604.

## Ethics Statement

The studies involving human participants were reviewed and approved by the Internal Review Board of the University of Regensburg (ref.: 17-782_1-101). The participants provided their written informed consent to participate in this study.

## Author Contributions

FC, AA-A, AH, and AG conceived and designed the experiments. XM, AH, DA, KS, and DM performed the experiments. XM, AH, FC, AG, KS, K-AH, TM, WB, and EH analyzed and interpreted the data. FC and AA-A acquired the funding and supervised the study. FC, XM, and AH wrote the manuscript with input from all authors. All authors reviewed and approved the manuscript.

## Conflict of Interest

The authors declare that the research was conducted in the absence of any commercial or financial relationships that could be construed as a potential conflict of interest.

## Publisher’s Note

All claims expressed in this article are solely those of the authors and do not necessarily represent those of their affiliated organizations, or those of the publisher, the editors and the reviewers. Any product that may be evaluated in this article, or claim that may be made by its manufacturer, is not guaranteed or endorsed by the publisher.
